# Radiofrequency Ablation Provides Rapid and Durable Pain Relief for the Palliative Treatment of Lytic Bone Metastases Independent of Radiation Therapy: Final Results from the OsteoCool Tumor Ablation Post-Market Study

**DOI:** 10.1007/s00270-023-03417-x

**Published:** 2023-04-03

**Authors:** Jason Levy, Elizabeth David, Thomas Hopkins, Jonathan Morris, Nam D. Tran, Hamed Farid, Francesco Massari, William G. O’Connell, Alexander Vogel, Afshin Gangi, Peter Sunenshine, Robert Dixon, Nicolas Von der Höh, Sandeep Bagla

**Affiliations:** 1 Department of Interventional Radiology, Northside Hospital, Atlanta, GA 30342 USA; 2 Department of Vascular/Interventional Radiology, Sunnybrook Health Sciences Centre, Toronto, ON M4N 3M5 Canada; 3 Department of Anesthesiology, Duke University Medical Center, Durham, NC 27710 USA; 4 Department of Radiology, Mayo Clinic, Rochester, MN 55905 USA; 5 Department of Neurooncology, Moffitt Cancer Center, Tampa, FL 33612 USA; 6 Department of Interventional Neuroradiology, St. Jude Medical Center, Fullerton, CA 92835 USA; 7 Department of Radiology, University Massachusetts Memorial Medical Center, Worcester, MA 01655 USA; 8 Department of Radiology, Emory University, Atlanta, GA 30309 USA; 9 Department of Radiology, Renown Regional Medical Center, Reno, NV 89434 USA; 10 Department of Imagerie Interventionnelle, Hôpitaux Universitaires de Strasbourg - Nouvel Hôpital Civil, 67091 Strasbourg, France; 11 Department of Diagnostic Radiology, Vascular Interventional Radiology, Banner - University Medical Center, Phoenix, AZ 85006 USA; 12 Department of Radiology, University of Arkansas for Medical Sciences, Little Rock, AR 72205 USA; 13 Department of Orthopedics, Trauma Surgery and Plastic Surgery, Universitaetsklinikum Leipzig, 4103 Leipzig, Germany; 14 Department of Diagnostic and Vascular and Interventional Radiology, Prostate Centers USA, LLC, Falls Church, VA 22043 USA

**Keywords:** Ablation, RFA, Skeletal-related events, Osseous metastases, Radiation-induced fracture

## Abstract

**Purpose:**

The OsteoCool Tumor Ablation Post-Market Study (OPuS One) was a prospective, multi-national, single-arm study to investigate safety and effectiveness of radiofrequency ablation (RFA) for palliation of painful lytic bone metastases with 12 months of follow-up. RFA has demonstrated effective palliation of osseous metastases in small clinical studies with short-term follow-up; however, a long-term assessment with robust subject numbers is lacking.

**Materials and Methods:**

Prospective assessments were conducted at Baseline, 3 days, 1 week, and 1, 3, 6, and 12-months. Pain and quality of life were measured prior to RFA and postoperatively using the Brief Pain Inventory, European Quality of Life—5 Dimension, and European Organization for Research and Treatment of Cancer Care Quality of Life Questionnaire for palliative care. Radiation, chemotherapy and opioid usage, and related adverse events were collected.

**Results:**

206 subjects were treated with RFA at 15 institutions in OPuS One. Worst pain, average pain, pain interference and quality of life significantly improved at all visits starting 3 days post-RFA and sustained to 12 months (*P* < 0.0001). Post hoc analysis found neither systemic chemotherapy nor local radiation therapy at the index site of RFA influenced worst pain, average pain, or pain interference. Six subjects had device/procedure-related adverse events.

**Conclusion:**

RFA for lytic metastases provides rapid (within 3 days) and statistically significant pain and quality of life improvements with sustained long-term relief through 12 months and a high degree of safety, independent of radiation.

**Level of Evidence: 2b, Prospective, Non-Randomized, Post-Market study:**

This journal requires that authors assign a level of evidence to each article. For a full description of these Evidence-Based Medicine ratings, please refer to the Table of Contents or the online Instructions to Authors www.springer.com/00266.

**Supplementary Information:**

The online version contains supplementary material available at 10.1007/s00270-023-03417-x.

## Background

Two-thirds of patients with osseous metastatic cancer report pain that alters quality of life [1, 2]. Additionally, osseous metastases can cause skeletal-related events (SRE) including pathologic fractures or neurologic injury leading to severe morbidity [3]. Lytic osseous metastases result in more SREs than blastic [4].

Treatment is focused on pain relief, reduction of SREs, and improvement in quality of life. External beam radiation therapy (EBRT) is considered the standard of care for symptomatic patients. Lytic metastases provide challenges for EBRT as they are more likely to cause radiation-induced fractures [5, 6].

Small prospective studies on percutaneous RFA have demonstrated pain relief [7–10] and decreased opioid use [9]. These studies had few patients with limited follow-up and many patients were also treated with radiation or radiation use was not reported [7–10]. Bagla et al. previously had the largest number of patients at 50 in a prospective single-arm study [7]. Follow-up in these studies was mostly short term with only 17 patients assessed past 6 months in the four studies combined [7–10]. In addition, all four of these prospective studies excluded patients with posterior wall involvement in the spine. In real-world clinical situations, the referral pattern for ablation often includes tumors with posterior element involvement [7–10]. A large prospective, multicenter study in a real-world setting on palliative skeletal RFA with longer-term follow-up is lacking. Separating the potential radiation effects from RFA treatment of patients is also a crucial element that has not been investigated.

OPuS One is a prospective multicenter study designed to evaluate the effectiveness and safety of RFA for painful metastatic lytic lesions. Results from the first 100 patients followed up to 6 months after RFA treatment have been published [11]. This manuscript presents the full cohort (206 subjects) followed for up to 12 months, with post hoc analyses to assess the potential impact of systemic chemotherapy and radiation at the index site on outcomes.

## Methods

### Study Design

**Fig. 1:**
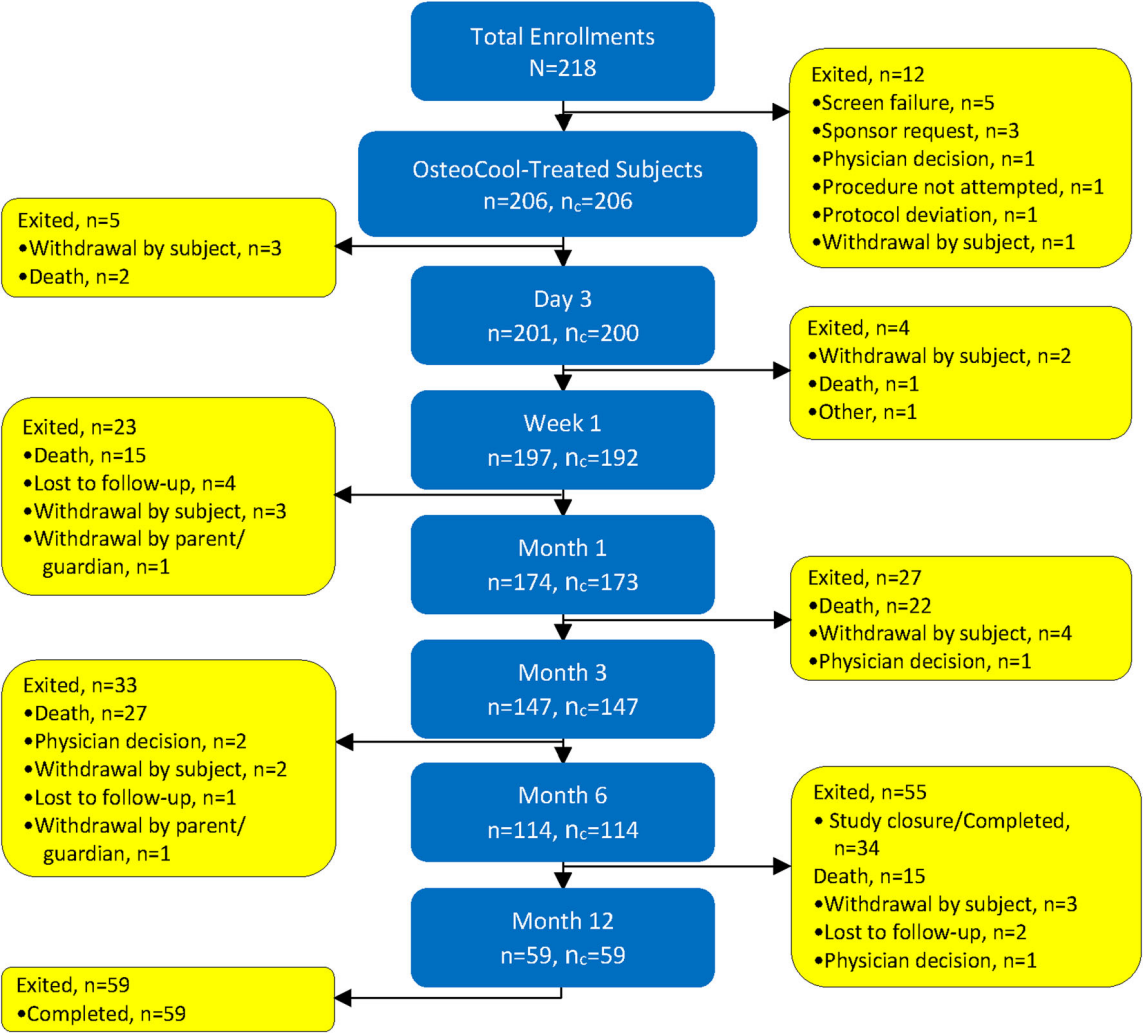
Subject disposition. *n*, number of subjects who were followed at the visit or at a later visit. *n*_c_, number of subjects who completed the visit

Two hundred eighteen (218) patients at 15 international centers were enrolled in a prospective, post-market, open label study—OsteoCool Tumor Ablation Post-Market Study (OPuS One)—between September 2017 through February 2020. The full study protocol is available online (NCT03249584).^1^ Patients were required to have worst pain ≥ 4/10 by Visual Analog Scale (VAS) within 24 h localized to the target site. Lesions were osteolytic or mixed osteolytic and osteoblastic in the thoracic and/or lumbar vertebral body(ies), periacetabulum, iliac crest, and/or sacrum. Exclusion criteria included: pure osteoblastic tumors, worst pain < 4/10 (VAS) in the last 24 h, more than two painful sites requiring treatment, or Karnofsky score [12] < 40. Figure 1 demonstrates the number of patients completing each follow-up visit. The most common reasons for discontinuation were death 82 (56%) and 34 (23%) subjects were discontinued after the 6 months visit due to early study closure by the sponsor.

### Ethics

Per the Helsinki Declaration, the Clinical Investigation Plan (CIP), Informed Consent Forms, and associated materials were each approved by the local Institutional Review Board (IRB) or Ethics Committee (EC). All patients provided informed written consent before participating in this study.

### RFA Procedure

Ablation of the targeted tumor(s) was performed using the OsteoCool™ RFA System (Medtronic Sofamor Danek, Memphis, TN). Target tumors were accessed using an 8, 10, or 13-gauge introducer cannula. At the completion of RFA, polymethylmethacrylate (PMMA) augmentation, if utilized, was injected through the same bone access cannula.

### Clinical Follow-up

Follow-up assessments occurred post-procedure at 3 days, 1 week, and 1, 3, 6 and 12-months. Subjects completed validated questionnaires to measure their pain, quality of life, and function. The Brief Pain Inventory (BPI) short form [13] is self-administered with 12 questions. Subjects rated their average and worst pain at the targeted area(s) in the last 24 h. A minimal clinically important difference (MCID) in pain, as measured by the BPI, was defined by ≥ 2-point change from baseline to post-procedure follow-up as previously established [14].

Quality of life measures included The European Quality of Life – Five Dimensions (EQ-5D) [15] and The European Organization for Research and Treatment of Cancer Care Quality of Life Questionnaire for palliative care (EORTC) [16]. Data were collected on transdermal and/or oral narcotics and then converted into OMED (oral morphine equivalent dose) [17]. Other treatments including radiation and procedure-related adverse events were also collected at baseline and at each follow-up visit.

### Statistical Analysis

Change in outcomes from baseline was tested for statistical significance. Statistical testing outside the primary and secondary objective (reported previously [11]) is considered post hoc. The normality of the change in outcome from baseline was tested by Shapiro–Wilk (SW) test. When *P* value was ≤ 0.05 from SW test, Wilcoxon signed-rank test was used; otherwise, paired *t* test was used. Also, post hoc was the decision to use the same test across all time points within an assessment. If the data at any of the timepoints are non-normal, a Wilcoxon signed-rank was applied; otherwise, a *t* test was used.

To evaluate the potential relevance of chemotherapy or radiation therapy at the site of RFA treatment on patient outcome measures (BPI worst pain, BPI average pain, BPI interference, and EQ-5D index), post hoc linear mixed modeling analysis was performed after adjusting for the following covariates: follow-up visit, baseline outcome measure, age, gender and days between baseline visit and RFA procedure. SAS software (version 9.4; SAS Institute, Cary, NC) was used for all analyses.

## Results

### Procedure

Although not required per protocol, PMMA augmentation was performed in 257 (97%) of procedures. Table 1 summarizes patient characteristics for the treated analysis set (206 subjects) and Table 2 provides the details of the ablation (264 procedures).

**Table 1:** Patient and tumor characteristics

	Treated analysis Set (*N* = 206)	
	Value	%
*Patients*		
Enrolled	218	–
Treated	206	–
*Sex*		
Female	113	55
Male	93	45
*Age*		
Mean (years)	63.7	–
Range	21–90	–
*Primary cancer*		
Breast	47	23
Lung	47	23
Gastrointestinal^a^	29	14
Kidney	21	10
Prostate	15	7
Liver	6	3
Skin	4	2
Thyroid	4	2
Endometrium	3	2
Lymph node	3	2
Bladder	2	1
Benign bone tumor	1	1
Bone	1	1
Non-cancerous^b^	1	1
Other	22	11
*Metastatic tumor location*		
Thoracic	88	43
Lumbar	78	38
Lumbar and thoracic	18	9
Periacetabulum^c^	7	3
Sacrum	7	3
Iliac crest	2	1
Lumbar and iliac crest	2	1
Sacrum and iliac crest	1	1
Lumbar and sacrum	1	1
Periacetabulum^c^ and sacrum	1	1
Thoracic and sacrum	1	1
*Procedure sites per subject*		
One metastatic lesion treated	151	73
Two metastatic lesions treated	52	25
Three metastatic lesions treated^d^	3	2
*Current treatments at baseline*^e^		
0	53	26
1	77	37
2	53	26
3	19	9
4	4	2
*Type of treatments at baseline*^f^		
Osteoporosis medications	78	38
Chemotherapy	70	34
Steroids	51	25
Antibody therapy	22	11
Immunotherapy	20	10
Radiation therapy	11	5
Surgical procedures	4	2

^a^Gastrointestinal includes colon, rectosigmoid, esophagus, gastrointestinal system, and pancreas

^b^Determined by biopsy after RFA

^c^RFA in the periacetabulum is on-label for OsteoCool; However, per protocol, investigators were free to deliver PMMA cement to RFA-treated sites at their discretion and the delivery of cement to the periacetabulum is off-label

^d^Deviations documented for treating 3 sites

^e^Before RFA

^f^Subjects may report more than one treatment, concomitant treatments

**Table 2:** Tumor radiofrequency procedure characteristics

	Procedure analysis set (*N* = 264)	
	Value	%
*Image guidance*		
Fluoroscopy	172	83
CT	28	14
Other^a^	6	3
*Anesthesia*		
General	105	51
Local conscious sedation	65	32
Monitored anesthesia care	36	17
*Procedure time*		
Mean (h)	1.1	–
Range	0.3–3.5	–
*Ablation number within target sites*^b^		
1 ablation	205	78
2 ablations	50	19
3 ablations	4	2
4 ablations	2	1
5 ablations	2	1
*RFA Approach, Vertebral*		
Vertebral ablation	240	–
Bilateral (2 probes)	198	83
Unilateral (1 probe)	42	18
*RFA Approach, Other*^c^		
Other locations	24	–
1 probe	7	29
2 probes	12	5
3 probes	2	8
4 probes	3	13
*Cement augmentation*		
Yes	257	97
No	7	3
*Cementoplasty type*		
Kyphoplasty	169	66
Vertebroplasty^d^	74	28
Cementoplasty	13	5
Other^e^	1	0
*Technical success*		
Yes	262	99
No	2	1

^a^CT and fluoroscopy

^b^For 1 target site, the ablation could not be conducted due to bone access issues

^c^Iliac crest, periacetabulum, sacrum

^d^Includes *n* = 20 “vertebral augmentation” cementoplasties

^e^Balloon kyphoplasty in acetabulum

### Pain Relief

**Fig. 2:**
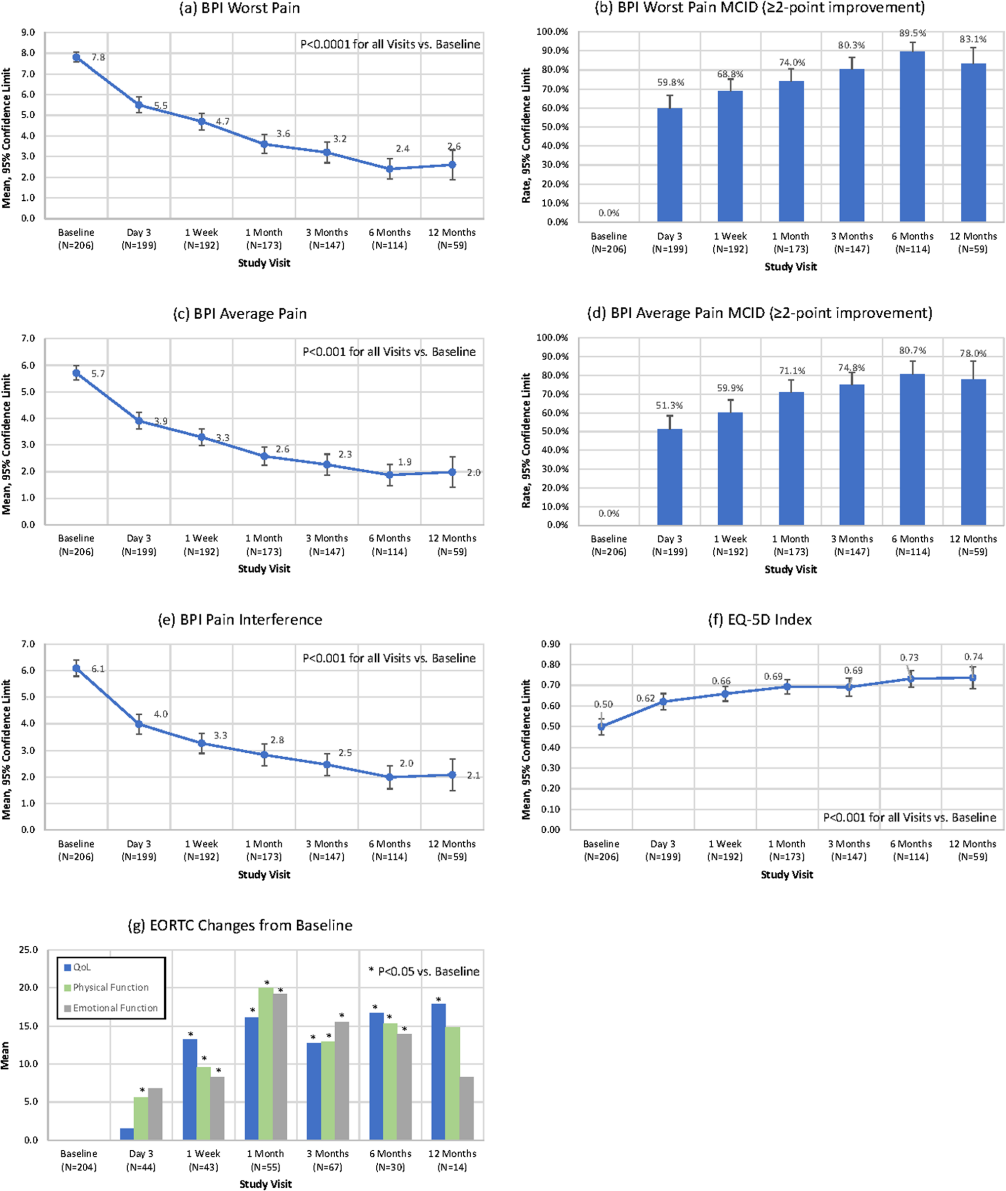
BPI Pain scores and QoL measures of patient outcomes for all RFA-treated subjects. **A** Change in BPI Worst Pain over time. **B** Percent of patients with a ≥ 2-point improvement in BPI Worst Pain over time. **C** Change in BPI Average Pain over time. **D** Percent of patients with a ≥ 2-point improvement in BPI Average Pain over time. **E** Change in BPI Pain Interference over time. **F** Change in EQ-5D Index over time. **G** Change in EORTC domain scores vs. Baseline over time

Following RFA, patients experienced significant improvement in worst pain and average pain at 3 days sustained up to twelve months (Fig. 2a, c). Over half (59.8%) of patients reported a MCID in worst pain at the targeted treatment site(s) 3 days post-ablation (Fig. 2b). A complete or partial response (as defined by the International Consensus on Palliative Radiotherapy [3]) was achieved in 74% of patients at 12 months (Table 3).

**Table 3:** Overall therapy response rate as defined using The International Consensus on Palliative Radiotherapy [3]

	3 days (*N* = 200)	1 week (*N* = 192)	1 month (*N* = 173)	3 months (*N* = 147)	6 months (*N* = 114)	12 months (*N* = 59)
Complete response (%)^a^	3	8	19	27	30	27
Partial response (%)^b^	50	50	42	37	40	47
Pain progression (%)^c^	13	11	6	10	4	3
Indeterminate response (%)^d^	34	31	33	27	26	22
Complete or partial response (%)	53	58	61	63	70	74

^a^Pain score of 0 at treated site with no concomitant increase in OMED

^b^Pain reduction of ≥ 2 (out of 10) without OMED increase, or OMED reduction of ≥ 25% from baseline without increase in pain

^c^Increase in pain score ≥ 2 (out of 10) from baseline at the treated site with stable OMED, or an increase of ≥ 25% in OMED compared to baseline with stable pain score or 1 point above baseline

^d^Any response that is not captured by the prior 3 response categories

### Quality of Life

Following RFA, the mean EQ-5D quality of life index improved significantly at every time point from 3 days to 12 months (Fig. 2f). There was a significant improvement in EORTC in all three domains (quality of life, physical and emotional functioning) at all visits (*P* < 0.05) except 3 days for quality of life and emotional functioning and 12 months for physical and emotional functioning (Fig. 2g). The degree of pain interference with patient’s functionality, as assessed by the BPI, showed significant improvement post-RFA from baseline (*P* < 0.0001 for all visits) (Fig. 2e).

### Oral Morphine Equivalent Dose (OMED)

More subjects decreased their OMED than increased their OMED at all visits, with the percentage of subjects with decreased OMED ranging from 34 to 51% (*Table S2* in Supplemental Digital Content).

### Radiation Therapy, Chemotherapy and Outcomes

From Baseline through the end of follow-up, 166/206 (81%) of subjects never received radiation therapy at the index site of RFA and 81/206 (39%) were off systemic chemotherapy (Table 4). After adjusting for covariates, post hoc linear mixed modeling did not find systemic chemotherapy or local radiation therapy at the index site of RFA to be significant predictors of worst pain, average pain, or pain interference on the BPI score for up to 12 months (Tables S2–S6 in Supplemental Digital Content). Meanwhile, radiation therapy, but not chemotherapy, was found to have reduced improvement in EQ-5D index (*P* < 0.02). A post hoc sub-group analysis was also performed on radiation-naïve subjects through end of follow-up (166/206) and found significant improvements in BPI worst pain, BPI average pain, BPI interference, and EQ-5D index at each follow-up visit through 12-months (*P* < 0.001, Figs. S1–S4 in Supplemental Digital Content).

**Table 4:** Exposure to chemotherapy and radiation therapy at the site of RFA among subjects that received RFA

Therapy	Exposure	Baseline (*N* = 206)	Baseline through end of follow-up (*N* = 206)
Radiation	Yes	11 (5.3%)	40 (19.4%)
	No	195 (94.7%)	166 (80.6%)
Chemo	Yes	70 (34.0%)	125 (60.7%)
	No	136 (66.0%)	81 (39.3%)

### Adverse Events

Device, therapy, and/or procedure-related adverse events, in 6/206 patients (2.9%), were reported including, drug hypersensitivity, folliculitis, intramuscular hematoma, intra-abdominal fluid collection, pneumonia, and respiratory failure. The latter three adverse events were considered serious. A total of 82 deaths (40%) were reported during the study. All deaths were classified by the Clinical Events Committee and none were related to the device, therapy, or procedure but instead attributed to the natural course of disease. No post-procedure vertebral fractures at the treated site(s) were observed for the duration of the study.

## Discussion

The results of this large prospective study of percutaneous ablation treatment for osseous metastases add to evidence that percutaneous ablation is safe and effective [7–10, 18]. Bagla et al. demonstrated significant improvement in mean scores for pain, disability, and cancer-specific health-related quality of life as early as 3 days post-procedure [7]. Most other prospective ablation studies assessed response at 1 week but did not assess response as early as 3 days. Our results demonstrated improved worst pain, average pain, pain interference, EQ-5D, and EORTC physical functioning, all of which were statistically improved at every time point from 3 days to 12 months. The rapid improvement at 3 days is clinically important given that radiation takes 3–6 weeks to achieve palliation [19]. Rapid improvement in pain and quality of life indices serves to maintain performance status, ability to stay on systemic protocol, and avoidance of the vicious cycle of pain [2].

Other studies have shown durable pain palliation from percutaneous ablation, but their clinical impact is limited by smaller numbers or shorter follow-up duration. Tanigawa et al. showed a 70% pain overall response rate in 33 patients, but only six patients were followed up to 12 months post-RFA [10]. Goetz et al. followed 43 patients up to 24 weeks (median 16 weeks) and demonstrated 95% overall response rate in significant pain relief, but only 12 patients were assessed at 24 weeks [9]. In the current study, 114 patients were followed up at 6 months and 59 patients were followed up to 12 months. At 12 months, there was an 83 and 78% clinically meaningful response rate in worst and average pain, respectively, and significant improvements in pain interference and EQ-5D index. Although this is not a comparative trial, EBRT, the current gold standard for pain palliation from osseous metastases, is limited in effectiveness with partial and complete response estimated at 60 and 33%, respectively [20]. Attempts to improve on palliation using SBRT failed to produce any meaningful differences in two prospective comparative trials [21, 22]. A recently published randomized controlled trial did demonstrate differences, but these were not evident until 3 months post-treatment and the SBRT arm suffered from radiation-induced fractures as well as pain flare at 1-month in 11 and 43% of subjects, respectively [23].

Complication rates reported for osseous RFA are low with multiple prospective and retrospective series reporting no SREs [7, 10, 18, 24]. In a retrospective review, Wallace et al. reported no major complications related to RFA and no instances of symptomatic cement extravasation despite a high-risk patient cohort similar to OPuS One including 89/110 (81%) of metastases involving the posterior vertebral body and/or pedicles [24]. In the current study, no post-procedure fractures at the ablation site(s) were reported despite all treated lesions having a lytic component. At the completion of RFA, 97% of the lesions were augmented with PMMA. PMMA has mechanical stabilization properties which protects against fractures. Most lytic metastases occur in axial weight loading bones including thoracolumbar spine and periacetabular locations, where mechanical stabilization is crucial [25]. Single-fraction EBRT, multi-fraction EBRT and SBRT all carry a risk of future fractures reported at 5–39%, so the protective effect of RFA with PMMA is clinically impactful [5, 26–30]. One of the biggest risks to develop radiation-induced fractures is having a significant lytic component [5]. Furthermore, despite high-risk lesions in our study, all of which were lytic and many of the spinal metastases involved the posterior vertebral body and/or pedicles, no neurovascular injuries occurred. In addition to the morbidity related to a fracture or neurologic injury, once a SRE occurs the prognosis for subsequent events and life expectancy worsens [31].

Other musculoskeletal ablation studies had higher rates of previous radiation use or did not report the numbers [7–9, 32]. Goetz reported 74% of patients were treated with radiation [9]. The relative lack of radiation treatment at baseline (5.3% [11/206]) and through post-RFA follow-up for up to 12 months (80.5% [166/206]) should be considered a strength of OPuS One. Post hoc linear mixed modeling of our final dataset did not find significant relationships between chemo or radiation therapy and worst pain, average pain or pain interference. The analysis demonstrated a reduction in EQ-5D improvement with radiation. This trend could be attributed to random variation due to small sample size in patients with radiation therapy or selection bias. Nevertheless, these results suggest that RFA with cement augmentation has the potential to provide pain relief and improved quality of life, with or without radiation therapy at the affected site.

One limitation of our study is the dropout rate. The most common cause of dropout was death from the underlying disease. This could be addressed with future studies moving RFA earlier in the care continuum. An additional limitation is the concurrent use of other therapies. Prior to the procedure the patients had chemotherapy (34%) or steroids (25%). While these agents may have provided some effect, the typical referral pattern for RFA is made when the pain is recalcitrant suggesting these agents failed to palliate [28]. Finally, 97% of the patients were augmented with PMMA so the impact of RFA versus PMMA is unclear. The rationale of combining the benefits of RFA for tumor control, reduction of osteoclast activity and biologic pain, with the administration of PMMA for mechanical stabilization has been previously described [33–35]. RFA performed alone in an axial weight loading bone without PMMA is unlikely to be studied in detail due to the risk of fracture. In addition, although some of the palliative effects in our cohort may be attributed to PMMA rather than RFA, a recent meta-analysis using machine learning concluded that RFA was effective for palliation regardless of the use of PMMA [36].

In conclusion, the study demonstrated safe, rapid and durable palliation with no SREs. Given that many of the advantages noted in this study address the more commonly seen disadvantages of radiation including time to pain relief and post-radiation fractures, future investigation to evaluate the benefits of pairing RFA-assisted PMMA augmentation with radiation in the palliative setting is warranted.

## Supplementary Information

Below is the link to the electronic supplementary material.

Supplementary file1 (DOCX 80 KB)

## Footnotes

1 https://www.clinicaltrials.gov/ProvidedDocs/84/NCT03249584/Prot_SAP_000.pdf

## Declarations

### Human or Animal Participants

All procedures performed in studies involving human participants were in accordance with the ethical standards of the institutional and/or national research committee and with the 1964 Helsinki declaration and its later amendments or comparable ethical standards.
