# Supplementary material for: Radiofrequency Ablation Provides Rapid and Durable Pain Relief for the Palliative Treatment of Lytic Bone Metastases Independent of Radiation Therapy: Final Results from the OsteoCool Tumor Ablation Post-Market Study
Source: Cardiovasc Intervent Radiol. 2023 Apr 3;46(5):600–9. doi: 10.1007/s00270-023-03417-x (PMC10156864; doi:10.1007/s00270-023-03417-x)
Supplement: Supplementary file 1 — Supplementary file1 (DOCX 80 KB) [file 270_2023_3417_MOESM1_ESM.docx]

# Table S1. Recalculation of the Primary objective analysis: change in worst pain at three months for vertebral subjects.

The primary objective was to evaluate if there was an improvement in the worst pain score from baseline to 3 months post RFA in subjects treated for metastatic lesions in only the thoracic and/or lumbar vertebral body(ies). The primary objective hypothesis testing was completed on April 11, 2019 as planned in the Clinical Investigation Plan/Statistical Analysis Plan and has been previously reported as being met [1]. Re-calculation of the primary objective using the final data set continued to demonstrate significant improvement from baseline to 3 months post RFA in worst pain score in subjects treated for metastatic lesions in the thoracic and/or lumbar vertebral body(ies) (N = 132, P < 0.0001) (Table S1 in Supplementary Information), confirming the robustness of the previously reported hypothesis test with data from 42 subjects. Sensitivity analyses confirmed that the change in pain from baseline to three months is robust to missing data, as the analyses demonstrated results consistent with those of only subjects with complete data. CI, confidence interval; LOCF, last observation carried forward.

| Analysis Method | | N | Mean | | Change | | *P* Value |
| --- | --- | --- | --- | --- | --- | --- | --- |
|  |  |  | Baseline | 3 months | Mean | 95% CI |  |
| Primary (completers) | | 132 | 7.5 | 3.2 | -4.3 | -4.9 to -3.7 | <0.0001 |
| Sensitivity analyses | Multiple imputation | 184 | 7.8 | 3.2 | -4.5 | -5.1 to -3.9 | <0.0001 |
|  | LOCF | 184 | 7.8 | 3.9 | -3.9 | -4.4 to -3.5 | <0.0001 |

# Table S2: Pain, quality of life, and additional outcomes at baseline through 12 months follow-up.

Note: 95% CIs are the 95% 2-sided CIs for the change from baseline.

|  | | Treated Analysis Set | | | | | | |
| --- | --- | --- | --- | --- | --- | --- | --- | --- |
|  |  | Baseline | 3 days | 1 week | 1 month | 3 months | 6 months | 12 months |
| BPI Worst Pain | **n** | 206 | 199 | 192 | 173 | 147 | 114 | 59 |
|  | **Score, mean** | 7.8 | 5.5 | 4.7 | 3.6 | 3.2 | 2.4 | 2.6 |
|  | **Standard Deviation** | 1.7 | 2.8 | 2.8 | 3.0 | 3.1 | 2.6 | 2.8 |
|  | **Median** | 8.0 | 5.0 | 5.0 | 3.0 | 2.0 | 2.0 | 2.0 |
|  | ***P* value (wilcoxon signed rank test), vs baseline** | - | <.0001 | <.0001 | <.0001 | <.0001 | <.0001 | <.0001 |
|  | **95% CI of change** | - | ( -2.7, -1.9) | ( -3.4, -2.6) | ( -4.5, -3.5) | ( -4.9, -3.8) | ( -5.6, -4.5) | ( -5.8, -4.2) |
|  | **Subjects with ≥ 2 point change (%)** | - | 59.8 | 68.8 | 74.0 | 80.3 | 89.5 | 83.1 |
| BPI Average Pain | **n** | 206 | 199 | 192 | 173 | 147 | 114 | 59 |
|  | **Score, mean** | 5.7 | 3.9 | 3.3 | 2.6 | 2.3 | 1.9 | 2.0 |
|  | **Standard Deviation** | 2.0 | 2.2 | 2.2 | 2.3 | 2.4 | 2.1 | 2.2 |
|  | **Score, median** | 6.0 | 4.0 | 3.0 | 2.0 | 2.0 | 1.0 | 1.0 |
|  | ***P* value (wilcoxon signed rank test), vs baseline** | - | <.0001 | <.0001 | <.0001 | <.0001 | <.0001 | <.0001 |
|  | **95% CI of change** | - | ( -2.1, -1.4) | ( -2.7, -2.0) | ( -3.3, -2.5) | ( -3.6, -2.7) | ( -4.1, -3.1) | ( -4.4, -3.0) |
|  | **Subjects with ≥ 2 point change (%)** | - | 51.3 | 59.9 | 71.1 | 74.8 | 80.7 | 78.0 |
| BPI Pain Interference | **n** | 205 | 199 | 192 | 173 | 147 | 114 | 59 |
|  | **Score, mean** | 6.1 | 4.0 | 3.3 | 2.8 | 2.5 | 2.0 | 2.1 |
|  | **Standard Deviation** | 2.2 | 2.6 | 2.7 | 2.7 | 2.6 | 2.3 | 2.3 |
|  | **Score, median** | 6.1 | 3.9 | 2.9 | 2.3 | 1.7 | 1.0 | 0.9 |
|  | ***P* value (t test), vs baseline** | - | <.0001 | <.0001 | <.0001 | <.0001 | <.0001 | <.0001 |
|  | **95% CI of change** | - | ( -2.4, -1.7) | ( -3.1, -2.4) | ( -3.5, -2.7) | ( -3.8, -2.8) | ( -4.3, -3.2) | ( -4.7, -3.3) |
| EQ-5D index | **n** | 205 | 199 | 192 | 173 | 147 | 114 | 59 |
|  | **Score, mean** | 0.50 | 0.62 | 0.66 | 0.69 | 0.69 | 0.73 | 0.74 |
|  | **Standard Deviation** | 0.28 | 0.29 | 0.25 | 0.23 | 0.27 | 0.22 | 0.21 |
|  | **Score, median** | 0.57 | 0.70 | 0.72 | 0.72 | 0.75 | 0.75 | 0.75 |
|  | ***P* value (wilcoxon signed rank test), vs baseline** | - | <.0001 | <.0001 | <.0001 | <.0001 | <.0001 | <.0001 |
|  | **95% CI of change** | - | ( 0.08, 0.15) | ( 0.12, 0.19) | ( 0.13, 0.20) | ( 0.09, 0.19) | ( 0.13, 0.23) | ( 0.14, 0.28) |
| EORTC  Quality of Life | **n** | 204 | 44 | 44 | 55 | 67 | 30 | 14 |
|  | **Change from baseline, mean** | 0.0 | 1.5 | 13.2 | 16.1 | 12.7 | 16.7 | 17.9 |
|  | **Standard Deviation** | 0.0 | 31.3 | 31.0 | 29.6 | 35.4 | 27.7 | 28.8 |
|  | **Change from baseline, median** | 0.0 | 0.0 | 16.7 | 16.7 | 16.7 | 16.7 | 16.7 |
|  | ***P* value (wilcoxon signed rank test), vs baseline** | - | 0.8992 | 0.028 | <.0001 | 0.0105 | 0.0007 | 0.043 |
|  | **95% CI of change** | - | ( -8.0, 11.0) | ( 3.6, 22.7) | ( 8.1, 24.1) | ( 4.0, 21.3) | ( 6.3, 27.0) | ( 1.2, 34.5) |
| EORTC  Physical Functioning | **n** | 204 | 44 | 44 | 55 | 67 | 30 | 14 |
|  | **Change from baseline, mean** | 0.0 | 5.6 | 9.5 | 20.0 | 12.9 | 15.3 | 14.8 |
|  | **Standard Deviation** | 0.0 | 22.7 | 17.1 | 27.8 | 31.9 | 27.5 | 35.0 |
|  | **Change from baseline, median** | 0.0 | 3.3 | 0.0 | 20.0 | 6.7 | 20.0 | 20.0 |
|  | ***P* value (wilcoxon signed rank test), vs baseline** | - | 0.0379 | 0.0002 | <.0001 | 0.0022 | 0.0015 | 0.1113 |
|  | **95% CI of change** | - | ( -1.3, 12.5) | ( 4.2, 14.7) | ( 12.5, 27.5) | ( 5.1, 20.7) | ( 5.1, 25.6) | ( -5.4, 35.0) |
| EORTC  Emotional Functioning | **n** | 204 | 44 | 43 | 55 | 67 | 30 | 14 |
|  | **Change from baseline, mean** | 0.0 | 6.8 | 8.3 | 19.2 | 15.5 | 13.9 | 8.3 |
|  | **Standard Deviation** | 0.0 | 30.5 | 24.2 | 32.2 | 33.4 | 26.7 | 27.9 |
|  | **Change from baseline, median** | 0.0 | 0.0 | 0.0 | 16.7 | 8.3 | 12.5 | 0.0 |
|  | ***P* value (wilcoxon signed rank test), vs baseline** | - | 0.2533 | 0.042 | <.0001 | 0.0004 | 0.0089 | 0.2207 |
|  | **95% CI of change** | - | ( -2.4, 16.1) | ( 0.8, 15.9) | ( 10.6, 27.9) | ( 7.4, 23.7) | ( 3.9, 23.8) | ( -7.8, 24.5) |
| Overall response rate^2^ | **n** | - | 200 | 192 | 173 | 147 | 114 | 59 |
|  | **Complete response (%)** | - | 3 | 8 | 19 | 27 | 30 | 27 |
|  | **Partial response (%)** | - | 50 | 50 | 42 | 37 | 40 | 47 |
|  | **Pain progression (%)** | - | 13 | 11 | 6 | 10 | 4 | 3 |
|  | **Indeterminate response (%)** | - | 34 | 31 | 33 | 27 | 26 | 22 |
|  | **Complete or partial response (%)** | - | 53 | 58 | 61 | 64 | 70 | 74 |
| Narcotics | **n** | 206 | 200 | 192 | 173 | 147 | 114 | 59 |
|  | **Oral morphine equivalent dose (mg)** | 54.6 | 50.1 | 56 | 49.4 | 41.7 | 40.3 | 19.4 |
|  | **Decrease in mean morphine equivalent (%)** | - | 34 | 34 | 40 | 37 | 42 | 51 |
|  | **No change in mean morphine equivalent (%)** | - | 37 | 35 | 31 | 33 | 32 | 32 |
|  | **Increase in mean morphine equivalent (%)** | - | 30 | 31 | 29 | 30 | 25 | 17 |

# Table S3: Linear Mixed Model for the effect of radiation therapy and chemotherapy on BPI Worst Pain Scores.

The final dataset from Opus One was used for this analysis: N=206 RFA-treated subjects with up to 12 months of follow-up.

|  | Radiation Therapy on BPI Worst Pain Score | | Chemotherapy on BPI Worst Pain Score |
| --- | --- | --- | --- |
| Independent Variables | **P-value** | | **P-value** |
| Follow-up Visit | <.0001 | | <.0001 |
| Baseline BPI Worst Pain Score | <.0001 | | <.0001 |
| Therapy Usage (baseline through follow up) |  | | |
| Radiation Therapy | 0.1612 | N/A | |
| Chemotherapy | N/A | 0.1338 | |
| Age | 0.4089 | | 0.4726 |
| Gender (Male/Female) | 0.5491 | | 0.5556 |
| Time between Baseline Assessment and RFA Procedure (days) | 0.5116 | | 0.4120 |

# Table S4: Linear Mixed Model for the effect of radiation therapy and chemotherapy on BPI Average Pain Scores.

The final dataset from Opus One was used for this analysis: N=206 RFA-treated subjects with up to 12 months of follow-up.

|  | Radiation Therapy on BPI Average Pain Score | | Chemotherapy on BPI Average Pain Score |
| --- | --- | --- | --- |
| Independent Variables | **P-value** | | **P-value** |
| Follow-up Visit | <.0001 | | <.0001 |
| Baseline BPI Average Pain Score | <.0001 | | <.0001 |
| Therapy Usage (baseline through follow up) |  | | |
| Radiation Therapy | 0.1271 | N/A | |
| Chemotherapy | N/A | 0.2902 | |
| Age | 0.4331 | | 0.4706 |
| Gender (Male/Female) | 0.6644 | | 0.6650 |
| Time between Baseline Assessment and RFA Procedure (days) | 0.7534 | | 0.6746 |

# Table S5: Linear Mixed Effects Model for the effect of radiation therapy and chemotherapy on BPI Interference Scores.

The final dataset from Opus One was used for this analysis: N=206 RFA-treated subjects with up to 12 months of follow-up.

|  | Radiation Therapy on BPI Interference Pain Score | | Chemotherapy on BPI Interference Pain Score |
| --- | --- | --- | --- |
| Independent Variables | **P-value** | | **P-value** |
| Follow-up Visit | <.0001 | | <.0001 |
| Baseline BPI Interference Pain Score | <.0001 | | <.0001 |
| Therapy Usage (baseline through follow up) |  | | |
| Radiation Therapy | 0.3216 | N/A | |
| Chemotherapy | N/A | 0.1683 | |
| Age | 0.8232 | | 0.9129 |
| Gender (Male/Female) | 0.5721 | | 0.5932 |
| Time between Baseline Assessment and RFA Procedure (days) | 0.4760 | | 0.3689 |

# Table S6: Linear Mixed Model for the effect of radiation therapy and chemotherapy on EQ-5D Index Scores.

The final dataset from Opus One was used for this analysis: N=206 RFA-treated subjects with up to 12 months of follow-up.

|  | Radiation Therapy on EQ5D Index Score | | Chemotherapy on EQ5D Index Score |
| --- | --- | --- | --- |
| Independent Variables | **P-value** | | **P-value** |
| Follow-up Visit | <.0001 | | <.0001 |
| Baseline EQ5D Index Score | <.0001 | | <.0001 |
| Therapy Usage (baseline through follow up) |  | | |
| Radiation Therapy | 0.0125 | N/A | |
| Chemotherapy | N/A | 0.7701 | |
| Age | 0.5178 | | 0.5220 |
| Gender (Male/Female) | 0.4963 | | 0.4745 |
| Time between Baseline Assessment and RFA Procedure (days) | 0.5619 | | 0.5839 |

# Figure S1. BPI Worst Pain Score for RFA-treated, Radiation-Naïve Subjects.

# Figure S2. BPI Average Pain Score for RFA-treated, Radiation-Naïve Subjects.

# Figure S3. BPI Pain Interference Score for RFA-treated, Radiation-Naïve Subjects.

# Figure S4. EQ-5D Index for RFA-treated, Radiation-Naïve Subjects.

# Figure S5. Proportion of RFA-treated, Radiation-Naïve Subjects with at least a 2-Point Improvement BPI Worst Pain Score.

# References

1. Levy J, Hopkins T, Morris J, et al. (2020) Radiofrequency Ablation for the Palliative Treatment of Bone Metastases: Outcomes from the Multicenter OsteoCool Tumor Ablation Post-Market Study (OPuS One Study) in 100 Patients. J Vasc Interv Radiol, 31(11):1745-1752. Doi:10.1016/j.jvir.2020.07.014

2. Chow E, Hoskin P, Mitera G, et al. (2012) Update of the international consensus on palliative radiotherapy endpoints for future clinical trials in bone metastases. Int J Radiat Oncol Biol Phys, 82(5):1730-1737. Doi:10.1016/j.ijrobp.2011.02.008
